# Influence of Weak Base Addition to Hole-Collecting Buffer Layers in Polymer:Fullerene Solar Cells

**DOI:** 10.3390/molecules22020262

**Published:** 2017-02-09

**Authors:** Jooyeok Seo, Soohyeong Park, Myeonghun Song, Jaehoon Jeong, Chulyeon Lee, Hwajeong Kim, Youngkyoo Kim

**Affiliations:** 1 Organic Nanoelectronics Laboratory and KNU Institute for Nanophotonics Applications (KINPA), Department of Chemical Engineering, School of Applied Chemical Engineering, Kyungpook National University, Daegu 41566, Korea; jyseo@knu.ac.kr (J.S.); msong@knu.ac.kr (M.S.); jh-jeong@knu.ac.kr (J.J.); lcyyeon@knu.ac.kr (C.L.); khj217@knu.ac.kr (H.K.); 2 Advanced Composites Materials Technical Center, Toray Advanced Materials Korea, Gumi-Si, Gyeongbook 39422, Korea; soohyeong@torayamk.com; 3 Priority Research Center, Research Institute of Advanced Energy Technology, Kyungpook National University, Daegu 41566, Korea

**Keywords:** polymer:fullerene solar cells, hole-collecting layer, PEDOT:PSS, weak base, aniline, pH, power conversion efficiency, stability

## Abstract

We report the effect of weak base addition to acidic polymer hole-collecting layers in normal-type polymer:fullerene solar cells. Varying amounts of the weak base aniline (AN) were added to solutions of poly(3,4-ethylenedioxythiophene):poly(styrenesulfonate) (PEDOT:PSS). The acidity of the aniline-added PEDOT:PSS solutions gradually decreased from pH = 1.74 (AN = 0 mol %) to pH = 4.24 (AN = 1.8 mol %). The electrical conductivity of the PEDOT:PSS-AN films did not change much with the pH value, while the ratio of conductivity between out-of-plane and in-plane directions was dependent on the pH of solutions. The highest power conversion efficiency (PCE) was obtained at pH = 2.52, even though all devices with the PEDOT:PSS-AN layers exhibited better PCE than those with the pristine PEDOT:PSS layers. Atomic force microscopy investigation revealed that the size of PEDOT:PSS domains became smaller as the pH increased. The stability test for 100 h illumination under one sun condition disclosed that the PCE decay was relatively slower for the devices with the PEDOT:PSS-AN layers than for those with pristine PEDOT:PSS layers.

## 1. Introduction

Polymer solar cells have been extensively studied because of their potential merits for making flexible plastic solar modules that can be easily rolled up like wallpaper [1,2,3,4]. Low-cost manufacturing processes are expected for polymer solar cells due to the potential of using continuous fabrication technology based on roll-to-roll printing processes employing plastic film substrates [2,3,4,5,6]. It is considered that it should be possible to make large-area plastic solar modules at room temperature because polymeric solutions are used for the continuous wet-coating processes [5,6]. In order to achieve such benefits, semiconducting polymers are of crucial importance in the construction of light-absorbing active layers [1,2,7,8,9,10].

The coming-out of polymer solar cells, exhibiting a viable power conversion efficiency (PCE) values since the organic bulk heterojunction (BHJ) concept was first reported, can be ascribed to the control of effective charge percolation nanomorphology in the polymer:fullerene BHJ layers by varying material compositions, solvents, annealing conditions, etc. [11,12,13,14,15,16,17,18]. Recently, the PCE of polymer:fullerene solar cells reached ca. 10%–11% in a single stack structure, while tandem structures delivered ca. 12%–13% PCE [19,20,21,22,23,24,25,26,27,28]. Such enhanced PCEs in single stack structures can be also attributed to the introduction of interlayers in the inverted-type devices [29,30,31,32].

In the case of normal-type devices, a hole-collecting buffer layer is necessary for effective hole collections from the active layers because the work function of typical transparent conducting oxides, for example, indium-tin oxide (ITO), makes large energy barriers for the transfer of holes from the p-type polymer components in the BHJ layers [2,33,34,35]. As a hole-collecting buffer layer, poly(3,4-ethylenedioxythiophene):poly(styrenesulfonate) (PEDOT:PSS) has been extensively used thanks to its work function range suitable for the effective hole collections [36,37,38]. However, the strong acidity of PEDOT:PSS has been pointed as one of the problems leading to the poor stability of devices, which has motivated various approaches to solve this issue, including the addition of strong bases [39,40,41,42].

In this work, we attempted to make the acidity of PEDOT:PSS milder by adding a weak base because strong bases can give rise to adverse effects including undesirable breaking of doped states between PEDOT and PSS components. Aniline (AN), as a weak base, has been added to the PEDOT:PSS solution, leading to the aniline-doped PEDOT:PSS (PEDOT:PSS-AN), which were then used for the preparation of the hole-collecting buffer layers in the normal-type solar cells with the BHJ layers of poly(3-hexylthiophene) (P3HT) and [6,6]-phenyl-C_61_-butyric acid methyl ester (PC_61_BM). The acidity of the PEDOT:PSS solutions was changed according to the aniline content, which in turn influenced the PCE of the P3HT:PC_61_BM solar cells.

## 2. Materials and Methods

P3HT (regioregularity = 92.2%; weight-average molecular weight = 59 kDa; polydispersity index = 2.32) and PC_61_BM (purity = 99.5%) were purchased from Rieke Metals (Lincoln, NE, USA) and Nano-C (Westwood, MA, USA), respectively. A blend of P3HT and PC_61_BM (P3HT:PC_61_BM = 1:1 by weight) was dissolved in chlorobenzene at a solid concentration of ~30 mg/mL. PEDOT:PSS (PH500, conductivity = ~500 S/cm) was received from Heraeus (Clevios PH 500, Hanau, Germany), to which aniline was added by varying the molar ratio up to 1.8 mol % with dimethyl sulfoxide (DMSO, 5 vol %). Indium-tin oxide (ITO)-coated glass substrates (sheet resistance= 10 Ω/□) were cleaned using acetone and isopropyl alcohol, dried, and treated by UV-ozone cleaning system (AH-1700, Ahtech LTS, Anyang-si, Korea) for 20 min. The PEDOT:PSS-AN layers (40 nm thick) were spin-coated on the ITO-glass substrates and thermally annealed at 230 °C for 15 min. Then the P3HT:PC_61_BM layers (ca. 85 nm) were spin-coated on the PEDOT:PSS layers that were coated on the ITO-glass substrates and soft-baked at 60 °C for 15 min. These samples were moved into a vacuum chamber for the deposition of lithium fluoride (LiF, ca. 1 nm) and aluminum (Al, 100 nm) through a metal shadow mask, defining the active area of 0.09 cm^2^ (note that the active area was cross-checked by the optical microscope measurement). All devices were subject to thermal annealing at 150 °C for 10 min.

The thickness of films including electrodes was measured using a thickness profiler (Alpha Step 200, Tencor Instruments, Milpitas, CA, USA). The in-plane (IP) conductivity of the PEDOT:PSS-AN films was measured using a four-probe measurement system equipped with an semiconductor parameter analyzer (4200-SCS, Keithley, Cleveland, OH, USA), while the out-of-plane (OOP) conductivity was obtained from the current-voltage (I-V) curves of the stacked devices (glass/ITO/PEDOT:PSS-AN/Al). The work functions of the PEDOT:PSS-AN films were measured using a photoelectron yield spectrometer (AC2, Riken-Keiki, Tokyo, Japan). The surface morphology was measured using an atomic force microscopy (AFM, Nanoscope IIIa, Digital Instruments, Santa Barbara, CA, USA). The solar cell performance of devices was measured by employing a specialized solar cell measurement system which integrates a solar simulator (A-class, air mass 1.5 G, 100 mW/cm^2^, Newport Oriel, Irvine, CA, USA), an electrometer (Model 2400, Keithley), and optical components. The one sun condition (100 mW/cm^2^) was adjusted using a calibrated reference cell (BS-520, Bunko-Keiki, Sharp Electronics, Tokyo, Japan). The external quantum efficiency (EQE) of devices were measured without a white bias using a home-built EQE measurement system equipped with a light source (tungsten-halogen lamp, 150 W, ASBN-W, Spectral Products, Putnam, CT, USA), a monochromator (CM110, Spectral Products), and a calibrated Si photodiode (818-UV, Newport, Irvine, CA, USA).

## 3. Results and Discussion

**Figure 1:**
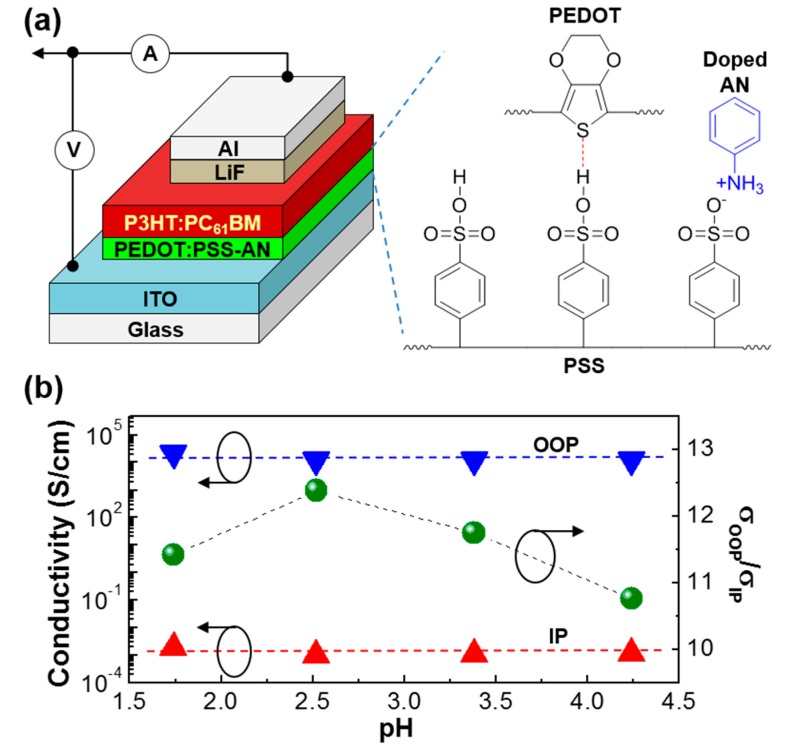
(**a**) Device structure for the P3HT:PC_61_BM solar cell with the polymer hole-collecting layer (PEDOT:PSS-AN); (**b**) Electrical conductivity and conductivity ratio (**σ**_OOP_/**σ**_IP_) of the PEDOT:PSS-AN films as a function of pH: Note that out-of-plane (OOP) and in-plane (IP) denote the directions perpendicular and parallel to the plane of films, respectively.

The device structure of the P3HT:PC_61_BM solar cells fabricated in this work is illustrated in Figure 1a, in which the hole-collecting PEDOT:PSS-AN layer is placed between the ITO electrode and the BHJ (P3HT:PC_61_BM) layer. As depicted on the right side in Figure 1a, aniline (AN) is thought to form additional doped states after reacting with the free sulfonic acid groups in the PSS component of PEDOT:PSS [43]. Interestingly, as shown in Figure 1b, the conductivity of the PEDOT:PSS-AN layers seems not to be so much changed even after aniline doping because no huge variation can be observed in the trend of conductivity for both in-plane (IP) and out-of-plane (OOP) directions. Note that the IP conductivity (**σ**_IP_, parallel to the film plane) was measured by employing a four-probe technique, while the OOP conductivity (**σ**_OOP_, normal to the film plane) was obtained from the I-V characteristics of diode-type devices (ITO/PEDOT:PSS-AN/Al). However, the conductivity ratios, OOP conductivity to IP conductivity (**σ**_OOP_/**σ**_IP_), was found to vary with the acidity (pH) of the solutions. The highest conductivity ratio was obtained at pH = 2.52 (AN = 1.2 mol %), which implies that the PEDOT:PSS-AN layer coated from the solution at pH = 2.52 can deliver the effective charge transport between the ITO electrode and the BHJ layer in the P3HT:PC_61_BM solar cells.

**Figure 2:**
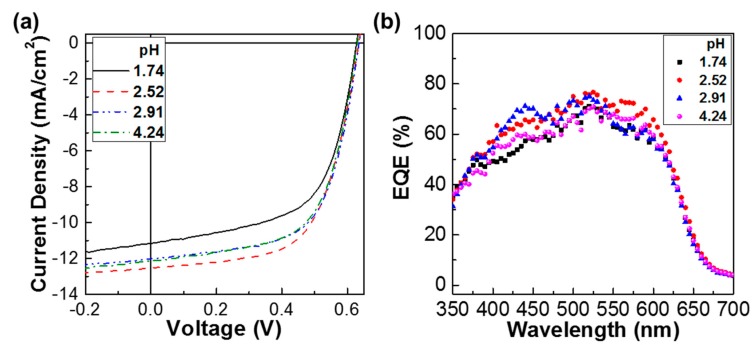
(**a**) Light J-V curves according to the pH of PEDOT:PSS layers for the P3HT:PC_61_BM solar cells under illumination with a simulated solar light (air mass 1.5G, 100 mW/cm^2^); (**b**) EQE spectra for the P3HT:PC_61_BM solar cells according to the pH of PEDOT:PSS layers.

Next, the current density–voltage (J-V) characteristics of the P3HT:PC_61_BM solar cells with the PEDOT:PSS-AN layers were measured under illumination with a simulated solar light (air mass 1.5 G, 100 mW/cm^2^). As shown in Figure 2, the light J-V curves were improved by the aniline-doped PEDOT:PSS (PEDOT:PSS-AN) layers. In particular, the light J-V curve at pH = 2.52 was better than any other J-V curves, implying an optimum aniline content for the best performance in the given device structure. It is worthy to note that the short circuit current density (J_SC_) was pronouncedly changed but the open circuit voltage (V_OC_) was only marginally varied according to the pH change. This result indicates that the charge transport in the devices might be considerably affected by the different pH of PEDOT:PSS-AN, whereas the photovoltage level was minimally influenced by the pH change.

**Figure 3:**
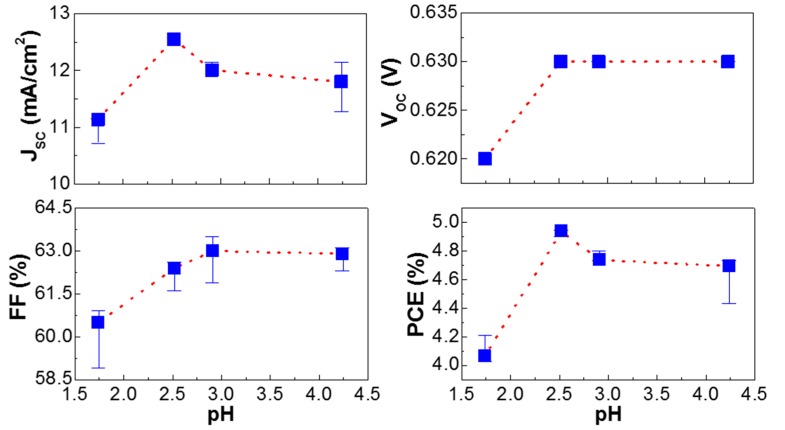
Solar cell parameters as a function of pH for the P3HT:PC_61_BM solar cells with the polymer hole-collecting layers (PEDOT:PSS and PEDOT:PSS-AN). All data were averaged from more than 20 devices.

The detailed trend of solar cell parameters is plotted in Figure 3 and Table 1. As briefly discussed in Figure 2, the highest J_SC_ value was obtained at pH = 2.52 (aniline content = 1.2 mol %). However, J_SC_ was gradually decreased after pH = 2.52 even though the J_SC_ value was still higher for the devices with the PEDOT:PSS-AN layers than the device with the pristine PEDOT:PSS layer. In contrast, V_OC_ was very slightly increased as the pH value increased. The fill factor (FF) was also gradually increased with the pH value, which is somewhat different from the trend of J_SC_. This can be explained by the continuously decreased series resistance (R_S_) trend and the successively increased solar cell shunt resistance trend, which can induce the efficient charge transport.

As a consequence, the PCE trend was similar to the J_SC_ trend because of the dominant influence of J_SC_ compared to other parameters. This result supports that the aniline addition (doping) has a critical influence on the charge collection characteristics in the present normal-type P3HT:PC_61_BM solar cells.

**Figure 4:**
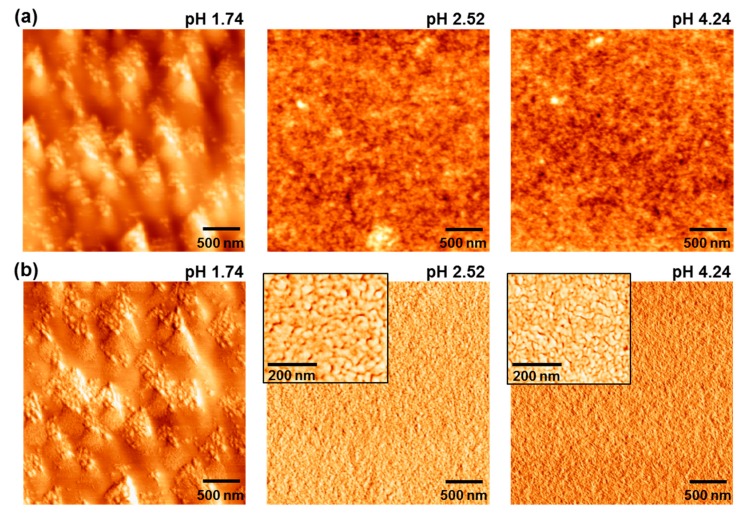
AFM images (**a**: height-mode, **b**: phase mode) for the pristine PEDOT:PSS (pH = 1.74) and PEDOT:PSS-AN layers (pH = 2.52 and 4.24) coated on the ITO-glass substrates. The scan size of images was 3 μm × 3 μm (inset: 1 μm × 1 μm).

In order to briefly understand the performance change according to the pH of PEDOT:PSS solutions with aniline, the surface morphology of the PEDOT:PSS-AN layers was examined with atomic force microscopy (AFM). As shown in Figure 4, both big and small domains (grains) were measured for the pristine PEDOT:PSS layer (without aniline). However, very fine domains were formed for the PEDOT:PSS-AN layers, irrespective of pH (i.e., aniline content). Further investigation provides that the size of such domains became much finer as the pH increased. This result informs that the aniline molecules added to the PEDOT:PSS solutions might effectively react with the free sulfonic acid parts of PSS and then change the aggregation size of PEDOT:PSS domains in the solutions (note that the work function of PEDOT:PSS-AN layers was almost unchanged ca. 5.1–5.2 eV as measured using the photoelectron yield spectrometer).

**Figure 5:**
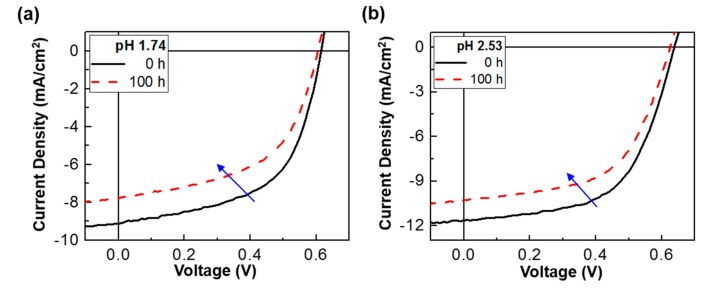
Light J-V curves for the P3HT:PC_61_BM solar cells with the polymer hole-collecting layers before and after continuous (100 h) illumination with a simulated solar light (air mass 1.5 G, 100 mW/cm^2^): (**a**) pristine PEDOT:PSS layer (pH = 1.74); (**b**) PEDOT:PSS-AN layer (pH = 2.52).

**Figure 6:**
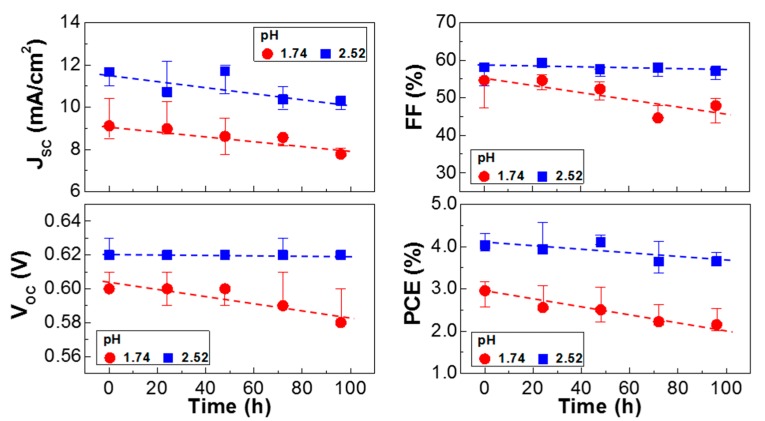
Solar cell parameters as a function of illumination time for the P3HT:PC_61_BM solar cells with the polymer hole-collecting layers under continuous (100 h) illumination with a simulated solar light (air mass 1.5 G, 100 mW/cm^2^): (red filled circles) pristine PEDOT:PSS layer (pH = 1.74), (blue filled squares) PEDOT:PSS-AN layer (pH = 2.52).

Taking into account the correlation between the device performance and the domain size, two small domains are considered to deliver an adverse effect in terms of charge transport as discussed on the conductivity ratio in Figure 1b. Finally, the stability of the P3HT:PC_61_BM solar cells with the pristine PEDOT:PSS layer (pH = 1.74) and the PEDOT:PSS-AN layer (pH = 2.52) was briefly tested under continuous (100 h) illumination with a simulated solar light (air mass 1.5 G, 100 mW/cm^2^). As shown in Figure 5, the light J-V curve was slightly changed after 100 h illumination for both pH = 1.74 and 2.52. The major change can be ascribed to the reduced J_SC_ in the presence of marginal decrease in V_OC_. The overall trend for >10 devices is plotted in Figure 6. The J_SC_ value was very similarly reduced with the illumination time for both devices, which may indicate the same origin on the charge collection in devices. Interestingly, the V_OC_ decay was very small for the devices with the PEDOT:PSS-AN layers, whereas it was relatively quite large for those with the pristine PEDOT:PSS layers. The similar trend was measured for the FF trend. Considering both V_OC_ and FF trends, it is shortly concluded that the reduced pH in the PEDOT:PSS-AN layers might have a positive influence on the prevention of degradation at the interfaces between the BHJ layer and the hole-collecting layer or between the ITO electrode and the hole-collecting layer. Consequently, the PCE decay was relatively slower for the devices with the PEDOT:PSS-AN layers than those with the pristine PEDOT:PSS layers.

## 4. Conclusions

The effect of aniline addition to the PEDOT:PSS layers was investigated by employing normal-type P3HT:PC_61_BM solar cells. The electrical conductivity in both OOP and IP directions was slightly changed with the pH variation but the conductivity ratio (OOP to IP directions) was varied with the pH value. The best solar cell performance was obtained at pH = 2.52, whereas the device performance was gradually decreased as the pH value increased further. The most influential parameter was J_SC_, while a minor contribution was given by both V_OC_ and FF. The surface nanomorphology revealed that the size of PEDOT:PSS domains became smaller with the addition of aniline (doping), and became much finer as the pH increased. The stability test for 100 h illumination under one sun condition showed that the PEDOT:PSS-AN layers are beneficial for preventing the decay of V_OC_ and FF in the present normal-type solar cells, even though the J_SC_ decay could not be prohibited owing to the intrinsic degradation inside the bulk heterojunction films.

## Figures and Tables

**Table 1:** Summary of solar cell parameters for the P3HT:PC_61_BM solar cells with the polymer hole-collecting layers (PEDOT:PSS and PEDOT:PSS-AN). Note that the parameter values were taken from the average ones in Figure 3 (air mass 1.5G, 100 mW/cm^2^).

Parameters	pH			
	1.74	2.52	2.91	4.24
**V_OC_ (V)**	0.62 (±0.01)	0.63 (±0.01)	0.63 (±0.01)	0.62 (±0.01)
**J_SC_ (mA/cm^2^)**	11.15 (±0.41) * (10.89)	12.54 (±0.09) * (12.15)	12.01 (±0.08) * (11.80)	12.14 (±0.52) * (11.67)
**FF (%)**	60.90 (±1.60)	62.60 (±0.80)	63.50 (±1.10)	62.9 (±0.60)
**PCE (%)**	4.21 (±0.14)	4.95 (±0.05)	4.80 (±0.07)	4.73 (±0.30)
**R_S_ (Ω cm^2^)**	110 (±17.0)	100 (±11.0)	90 (±11.0)	90 (±10.0)
**R_SH_ (kΩ cm^2^)**	5.7 (±0.01)	5.9 (±0.02)	10.3 (±0.02)	12.5 (±0.01)

* J_SC_ calculated from the EQE spectra in Figure 2b.
